# Identification of lymph node metastasis in pre‐operation cervical cancer patients by weakly supervised deep learning from histopathological whole‐slide biopsy images

**DOI:** 10.1002/cam4.6437

**Published:** 2023-08-10

**Authors:** Qingqing Liu, Nan Jiang, Yiping Hao, Chunyan Hao, Wei Wang, Tingting Bian, Xiaohong Wang, Hua Li, Yan zhang, Yanjun Kang, Fengxiang Xie, Yawen Li, XuJi Jiang, Yuan Feng, Zhonghao Mao, Qi Wang, Qun Gao, Wenjing Zhang, Baoxia Cui, Taotao Dong

**Affiliations:** ^1^ Cheeloo College of Medicine Shandong University Jinan City China; ^2^ Department of Pathology, School of Basic Medical Science, Cheeloo College of Medicine Shandong University Jinan City China; ^3^ Department of Pathology Qilu Hospital of Shandong University Jinan City China; ^4^ Department of Pathology Affiliated Hospital of Jining Medical University Jining City China; ^5^ Department of Medical Imaging Affiliated Hospital of Jining Medical University Jining City China; ^6^ Department of Obstetrics and Gynecology Jinan People's Hospital Jinan City China; ^7^ Department of Obstetrics and Gynecology Tai'an City Central Hospital Tai'an City China; ^8^ Department of Obstetrics and Gynecology Weifang People's Hospital Weifang City China; ^9^ Department of Obstetrics and Gynecology Women and Children's Hospital, Qingdao University Qingdao City China; ^10^ Department of Pathology KingMed Diagnostics Jinan City China; ^11^ Department of Obstetrics and Gynecology, Shandong Provincial Qianfoshan Hospital Shandong University Jinan City China; ^12^ Department of Obstetrics and Gynecology The Affiliated Hospital of Qingdao University Qingdao City China; ^13^ Department of Obstetrics and Gynecology Qilu Hospital of Shandong University Jinan City China

**Keywords:** cervical biopsy, cervical cancer, deep learning, histopathological, lymph node metastasis, WSI

## Abstract

**Background:**

Lymph node metastasis (LNM) significantly impacts the prognosis of individuals diagnosed with cervical cancer, as it is closely linked to disease recurrence and mortality, thereby impacting therapeutic schedule choices for patients. However, accurately predicting LNM prior to treatment remains challenging. Consequently, this study seeks to utilize digital pathological features extracted from histopathological slides of primary cervical cancer patients to preoperatively predict the presence of LNM.

**Methods:**

A deep learning (DL) model was trained using the Vision transformer (ViT) and recurrent neural network (RNN) frameworks to predict LNM. This prediction was based on the analysis of 554 histopathological whole‐slide images (WSIs) obtained from Qilu Hospital of Shandong University. To validate the model's performance, an external test was conducted using 336 WSIs from four other hospitals. Additionally, the efficiency of the DL model was evaluated using 190 cervical biopsies WSIs in a prospective set.

**Results:**

In the internal test set, our DL model achieved an area under the curve (AUC) of 0.919, with sensitivity and specificity values of 0.923 and 0.905, respectively, and an accuracy (ACC) of 0.909. The performance of the DL model remained strong in the external test set. In the prospective cohort, the AUC was 0.91, and the ACC was 0.895. Additionally, the DL model exhibited higher accuracy compared to imaging examination in the evaluation of LNM. By utilizing the transformer visualization method, we generated a heatmap that illustrates the local pathological features in primary lesions relevant to LNM.

**Conclusion:**

DL‐based image analysis has demonstrated efficiency in predicting LNM in early operable cervical cancer through the utilization of biopsies WSI. This approach has the potential to enhance therapeutic decision‐making for patients diagnosed with cervical cancer.

## 1 INTRODUCTION

In 2020, cervical cancer was identified as the fourth most prevalent cancer among females, with a total of 604,127 new cases reported globally and 341,831 deaths attributed to this malignancy.^1^, ^2^ The presence of lymph node metastasis (LNM) significantly heightens the risk of disease recurrence and cancer‐related mortality, thereby impacting treatment choices for patients with cervical cancer.^3^, ^4^, ^5^ The National Comprehensive Cancer Network (NCCN) guidelines continue to advocate for radical surgical interventions, specifically radical hysterectomy or radical cervical resection accompanied by pelvic lymph node dissection, as the recommended treatment for patients diagnosed with early‐stage cervical cancer. Nevertheless, the incidence of complications associated with systematic lymph node dissection, such as lymphatic cysts, infections, bleeding, and venous thrombosis, remains considerably high, significantly impacting the overall quality of life experienced by affected individuals. In cases of early‐stage cervical cancer, the incidence of LNM is estimated to be around 15.0%–20.0%, these patients are categorized as advanced cervical cancer, the combined treatment of radiotherapy and chemotherapy is highly recommended for advanced cervical cancer by NCCN. Hence, accurate preoperative assessment of lymph node status in cervical cancer patients is crucial, allowing patients with LNM to opt for chemoradiotherapy alone, avoiding the dual burden of surgery and chemoradiotherapy.^6^, ^7^, ^8^, ^9^ Additionally, accurate evaluation enables 80.0%–85.0% patients without LNM to avoid the complications associated with lymphadenectomy, thus enhancing the quality of life for cervical cancer patients and minimizing the risks associated with excessive treatment.

The identification of LNM was determined postoperatively through microscopic examination of all excised lymph nodes by pathologists. However, accurately predicting LNM prior to surgery remains challenging. Preoperative evaluation of lymph node status in patients with cervical cancer is typically conducted using contrast‐enhanced computed tomography (CT) and magnetic resonance imaging (MRI) scans.^10^ An enlargement of 1 cm or more in a lymph node is deemed abnormal and can be detected using either of these imaging techniques. However, it should be noted that lymph nodes can still be hyperplastic even when enlarged, and it is important to consider the possibility of metastatic disease spreading to lymph nodes smaller than 1 cm.^11^ A systematic review conducted revealed that CT scans were able to predict LNM with a sensitivity of 43%, while MRI scans only had a sensitivity of 60%. Consequently, both CT and MRI scans exhibit low sensitivity in predicting LNM for cervical cancer and may result in missed diagnoses. On the contrary, positron emission tomography (PET) demonstrated a sensitivity of 74.7% and a specificity of 97.6% for LNM. However, the widespread use of PET as a routine test is hindered by its high cost and the challenges it poses, particularly in low‐income countries.^12^, ^13^ The diagnostic accuracy of intraoperative assessments, such as sentinel node biopsies, for detecting LNM is reported to have an overall sensitivity of 77.4%. However, it should be noted that sentinel node biopsies necessitate the use of general anesthesia, making them a more invasive procedure.^14^, ^15^ Therefore, it is imperative to enhance the precision of preoperative assessments in order to determine the probability of LNM more accurately. This will enable healthcare professionals to make optimal therapeutic decisions for patients diagnosed with early‐stage cervical cancer.

In recent years, the utilization of digital slide scanners for the conversion of H&E‐stained pathological sections into whole‐slide images (WSIs) has gained prominence in the field of digitalization. The enhanced dependability of scanner technologies and the increased accessibility of WSIs have contributed to their growing prevalence. Simultaneously, the advent of faster networks and cost‐effective storage solutions has facilitated the emergence of computational pathology, thereby enabling computer‐aided diagnosis and the implementation of a digital workflow for pathologists.^16^, ^17^, ^18^ Furthermore, it is noteworthy that each WSI image possesses a substantial number of pixels and encompasses a vast amount of information. Consequently, the utilization of DL technology becomes imperative in order to delve deeper into the concealed information. This elucidates the effectiveness of auxiliary diagnostic tools that have been developed using WSIs and DL technology. Moreover, the advancement of DL has been further propelled by various competitions focused on the development of DL algorithms, such as CAMELYON16 and CAMELYON17. These competitions, held in 2016 and 2017 respectively, aimed to identify DL models capable of detecting and staging breast cancer metastases.^19^ Pathologists can enhance their precision and productivity through the utilization of diagnostic decision support tools.

Notably, deep learning‐based artificial neural networks (ANNs) have demonstrated potential in the detection of cervical cancer and LNM in various other cancers.^20^, ^21^ Employing a DL model, automatic diagnosis can be achieved using colposcopy images.^22^ Ehteshami et al. successfully developed a DL model for the identification of sentinel LNM.^23^ Clinical annotations frequently provide sufficient information to identify the target at the image level, such as the presence of cancer in WSIs. By employing a weakly supervised DL system, it becomes unnecessary to manually annotate cancer regions, as the system solely relies on reported diagnoses for training. This approach effectively circumvents the laborious and expensive process of pixel‐level manual annotations. In a study conducted by Campanella et al., utilizing weakly supervised deep learning, they achieved impressive areas under the curve exceeding 0.98 for classifying both benign and malignant cancers in breast, prostate, and basal cell carcinoma.^24^

The objective of this research was to create a deep learning‐based digital pathological feature extractor that can accurately forecast the probability of LNM using digitized H&E‐stained slides obtained from primary tumor biopsies conducted prior to surgery. The biopsied lesion acquired before the operation represents certain characteristics of the cancer. This prediction can serve as a valuable reference for the development of personalized treatment strategies, thereby mitigating the risks associated with unnecessary lymphadenectomy and its associated complications in cases where patients exhibit a very low likelihood of LNM. Conversely, in instances which patients are deemed to possess a significantly elevated risk of LNM or even metastasis in general, radical chemoradiotherapy is considered the preferred course of treatment.

## 2 MATERIALS AND METHODS

### 2.1 Patient cohorts

The patients' cohort with FIGO stage IA2‐IIA2 cervical cancer from Qilu Hospital of Shandong University, referred to as the Qilu cohort, was retrospectively included for the purpose of training, validation, and internal testing. Additionally, a large multicenter test cohort, referred to as the external cohort, was formed by including patients with FIGO stage IA2‐IIA2 cervical cancer from Affiliated Hospital of Jining Medical University, Jinan People's Hospital, Tai'an City Central Hospital, and Women and Children's Hospital of Qingdao University.

**FIGURE 1:**
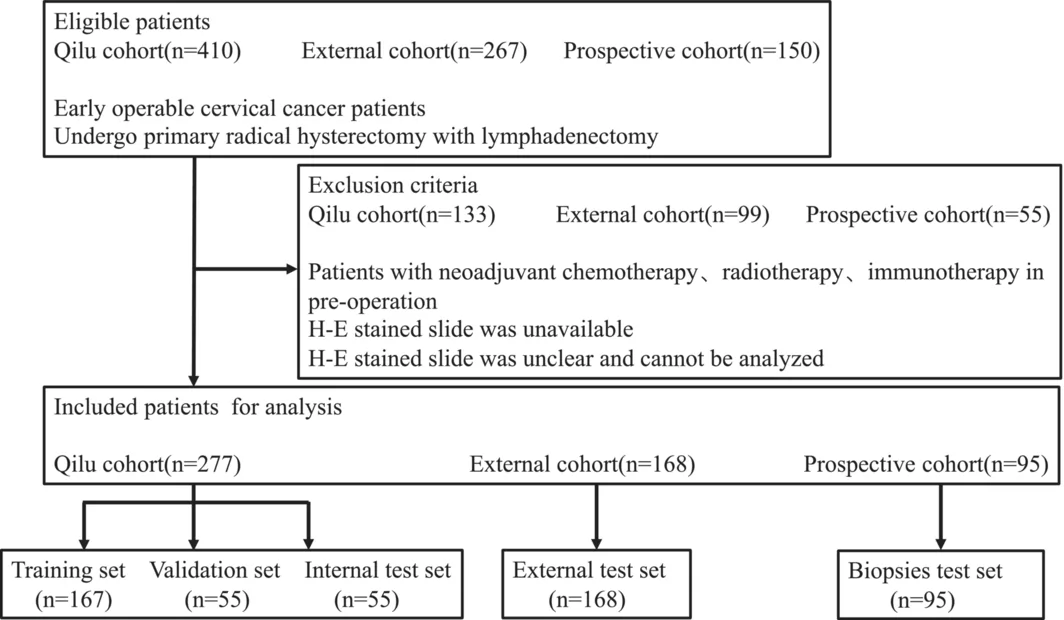
Data collect flow to create the training set, validation set and internal test set from Qilu cohort, as well as external test set from external cohort and biopsies test set from prospective cohort.

The study's inclusion criteria consisted of cervical cancer patients with FIGO stage IA2‐IIA prior to radical surgery. The eligible patients had squamous cell carcinoma, adenocarcinoma, or adeno‐squamous cell carcinoma as their pathological types. The surgical interventions included modified extensive hysterectomy or extensive hysterectomy plus pelvic lymph node dissection. Furthermore, the histopathological sections were required to be intact, and complete clinicopathologic data were necessary. Conversely, patients who had undergone neoadjuvant chemotherapy, radiotherapy, or immunotherapy prior to the operation were excluded from the study. H&E‐stained histological slides obtained from the Department of Pathology at both the Qilu cohort and external cohort hospitals were procured. For each patient, two slides of high quality, featuring cancerous tissue, were chosen. A pathologist with 15 years of clinical expertise from the Department of Pathology at Qilu Hospital, professor HCY, meticulously examined all the histological slides in preparation for subsequent digital scanning and analysis. A total of 277 patients (554 WSIs) from the Qilu cohort, diagnosed between 2010 and 2021, and 168 patients (336 WSIs) from the external cohort, diagnosed between 2015 and 2021, were included in our analysis. In order to enhance the clinical applicability of our DL model for the detection of LNM prior to surgery, we additionally incorporated 95 patients (190 WSIs) diagnosed with IA2‐IIA2 primary cervical cancer between 2017 and 2021 from the Qilu cohort as the prospective test group (referred to as the prospective cohort). In the preoperative cohort, we obtained excisional biopsies of their tissue, adhering to the same inclusion and exclusion criteria as above mentioned. The data collection process is illustrated in Figure 1.

In the Qilu cohort and prospective cohort, we retrospectively gathered CT or MRI radiography image reports of the patients, which were examined prior to the operation. If any lymph node enlargement was documented in the radiography image reports, suspicion of LNM arose, and subsequently, the accuracy of the radiography imaging examination was calculated.

### 2.2 Clinical‐pathological characteristics

The Qilu cohort was randomly divided into a training set (60%, *n* = 167, 40 LNM positive/127 LNM negative), a validation set (20%, *n* = 55, 13 LNM positive/42 LNM negative), and an internal test set (20%, *n* = 55, 13 LNM positive/42 LNM negative). The external cohort (*n* = 168, 43 LNM positive/125 LNM negative) and the prospective cohort (*n* = 95, 22 LNM positive/73 LNM negative) were used as the external test set and the biopsies test set, respectively. The characteristics of patients in each cohort are presented in Table 1. Statistical analyses were conducted using Student's t‐test and Pearson chi‐square analysis to assess the clinical‐pathological characteristics between the cohorts. A significance level of *p* < 0.05 was considered statistically significant.

**TABLE 1:** Descriptive characteristics of patients with cervical cancer in the Qilu cohort (*n* = 277), external cohort (*n* = 168), and prospective cohort (*n* = 95).

	Qilu cohort (*n* = 277)			External cohort (*n* = 168)	Prospective cohort (*n* = 95)
	Training set (*n* = 167)	Validation set (*n* = 55)	Internal testing set (*n* = 55)	External testing set (*n* = 168)	Biopsies test set (*n* = 95)
Age, years (mean [SD])	46 (10)	47 (10)	46 (11)	48 (11)	47 (11)
FIGO stage (%)					
IA2	7 (4.19)	2 (3.64)	2 (3.64)	5 (2.98)	9 (9.47)
IB1	60 (35.93)	19 (34.55)	20 (36.36)	47 (27.98)	29 (30.53)
IB2	51 (30.54)	17 (30.91)	17 (30.91)	52 (30.95)	30 (31.58)
IB3	25 (14.96)	9 (16.36)	8 (14.55)	36 (21.43)	9 (9.47)
IIA1	12 (7.19)	4 (7.27)	4 (7.27)	17 (10.12)	14 (14.74)
IIA2	12 (7.19)	4 (7.27)	4 (7.27)	11 (6.55)	4 (4.21)
Differentiation (%)					
High differentiation	58 (34.73)	18 (32.73)	18 (32.73)	68 (40.48)	26 (27.37)
Middle differentiation	47 (28.14)	16 (29.09)	16 (29.09)	81 (48.21)	46 (48.42)
Low differentiation	62 (37.13)	21 (38.18)	21 (38.18)	19 (11.31)	23 (24.21)
Histological (%)					
Squamous	54 (32.34)	17 (30.91)	17 (30.91)	113 (67.26)	43 (45.26)
Adenocarcinoma	100 (59.88)	33 (60.00)	33 (60.00)	51 (30.36)	46 (48.42)
Other	13 (7.78)	5 (9.09)	5 (9.09)	4 (2.38)	6 (6.32)
Tumor size, mm (mean (SD))	2.69 (0.58)	2.91 (0.62)	2.73 (0.65)	3.00 (0.72)	2.00 (0.47)
Depth of invasion (%)					
<1/2	93 (55.69)	31 (56.36)	31 (56.36)	65 (38.69)	52 (54.74)
≥1/2	74 (44.31)	24 (43.64)	24 (43.64)	103 (61.31)	43 (45.26)
Parametrial involvement (%)					
Yes	7 (4.19)	2 (3.64)	2 (3.64)	5 (2.98)	4 (4.21)
No	160 (95.81)	53 (96.36)	53 (96.36)	163 (97.02)	91 (95.79)
Lymphatic vascular invasion: LVI (%)					
Yes	54 (32.34)	17 (30.91)	17 (30.91)	42 (25.00)	31 (32.63)
No	113 (67.66)	38 (69.09)	38 (69.09)	126 (75.00)	64 (67.37)
Lymph node status diagnosed by radiography (%)					
Positive	37 (28.68)	13 (28.89)	13 (28.89)	—	14 (17.72)
Negative	92 (71.32)	32 (71.11)	32 (71.11)	—	65 (82.28)
Lymph node status diagnosed by histopathology (%)					
Positive	40 (23.95)	13 (23.64)	13 (23.64)	43 (25.60)	22 (23.16)
Negative	127 (76.05)	42 (76.36)	42 (76.36)	125 (74.40)	73 (76.84)

### 2.3 H&E‐stained slide scanning

The slides were scanned using a Pannoramic MIDI II scanner (3DHISTECH, Hungary) at ×20 equivalent magnification (0.23 μm per pixel) and digitized into mrxs format. Additionally, an isanCoreroAu scanner (Roche, America) at ×20 equivalent magnification (0.24 μm per pixel) was used to digitize the slides into BIF format. Prior to data processing, digital slides were manually examined for blur artifacts, and any unclear slides were rescanned.

### 2.4 WSIs preprocessing

**FIGURE 2:**
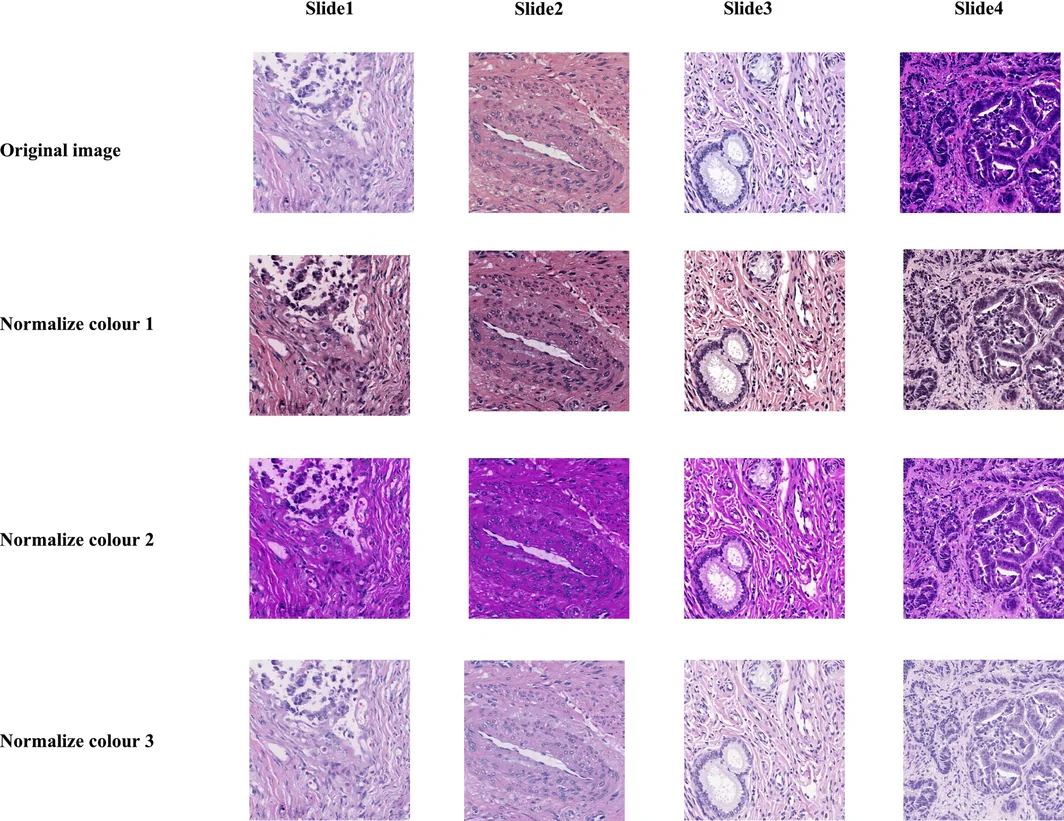
Illustration of the image color normalize procedure. The original image was color transformed using one color patterns by Macenko Normalizer algorithm, in this study, we adopted color pattern 3.

Initially, WSIs were categorized as either LNM (marked as 1) or non‐LNM (marked as 0) based on post‐operation histological reports. In order to encompass all potentially significant histopathologic regions, the complete WSIs were utilized as input, rather than manually outlining tumor areas. Nonoverlapping patches were generated by cropping WSIs at dimensions of 384 × 384 pixels using Pyvips (version 1.12.3). In accordance with the established definition of weakly supervised deep learning, the labeling of a patch as LNM positive (1) or LNM negative (0) was determined based on its origin within the WSI. To enhance computational efficiency, patches exhibiting image pixel variation below 500, denoting the absence of lesion tissues (referred to as white patches), were excluded. Subsequently, all patches underwent color normalization utilizing the MacenkoNormalizer method, as depicted in Figure 2, with the aim of enhancing the convergence of learning properties.^25^

### 2.5 Slide‐based DL predictor

**FIGURE 3:**
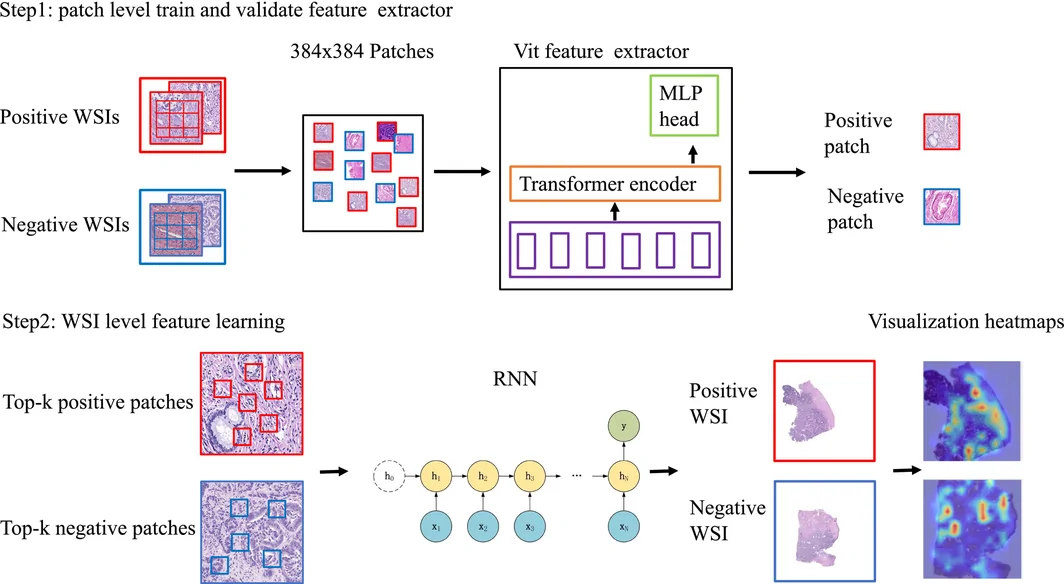
Overview of the proposed deep learning framework presented in this study. In step1: First, histopathology slides of primary tumor specimens were reviewed and annotated for lymph node status (positive and negative) by pathologists; second, histopathology slides were scanned and digitized into WSIs; third, WSIs were cropped into patches with 384 × 384 pixels at 20x for model development; fourth, the ViT network was trained to extract patches features, then classify each patch. In step2: We selected top‐K patches (K = 10, 50, 100, 500, 1000, 1500), then these K most representative patches in each slide were sequentially passed to the RNN to predict the final WSI‐level lymph node status. Ultimately, generated visualization heatmaps in each WSI. ViT, Vision transformer, RNN, Recurrent neural network, LNM, lymph node metastasis.

We selected Vision Transformer (Vit)^26^ and recurrent neural networks (RNN)^27^ as our DL architectures. Figure 3 provides a simplified overview of our pipeline. Our approach involved a two‐step process: first, training the patch features extractor ViT to learn features in histopathological images at the patch level, resulting in a representation of semantically rich patch‐level features. Subsequently, the predicted patch scores were used to determine the influence of patch features on the metastasis of the primary tumor to lymph nodes. Next, the patch index K denotes the ordinal value assigned to patch feature scores in descending order within each WSI, where a higher patch score corresponds to an increased likelihood of LNM. The term “Top‐K" (K = 10, 50, 100, 500, 1000, 1500) denotes the K patch features with the most elevated scores. These Top‐K‐ranked patches were utilized as input for an RNN. The RNN was employed for the purpose of aggregating the Top‐K patch features in order to generate a conclusive slide score. In the context of histopathological classification, the last fully connected layer of the RNN was substituted with a Softmax layer that produced a two‐dimensional vector. If the final slide score surpassed 0.5, the slide image was categorized as LNM positive; otherwise, it was classified as LNM negative. The DL model's performance was assessed by varying the patch index K, and the model exhibiting the most optimal performance was chosen.

### 2.6 Network training

The training procedure comprised of two sequential steps. Initially, we initialized the network by transferring weights from the vit_base_patch_384 dataset, with the exception of the last fully connected layer, which remained unaltered. Subsequently, all other layers were frozen and the model was trained using our histopathologic images. In the second step, we aimed to enhance the network's compatibility with the histopathological images by unfreezing the previously frozen layers and fine‐tuning them using our validation set. The modification and optimization of the parameters of the trainable layers were performed based on the evaluation of the cross‐entropy between the predictions and the ground truth labels. The initial learning rate was set to 1e‐4, weight decay was set to 1e‐4, and the Adam optimizer was utilized.^28^ Grid search was employed in each experiment to optimize the learning rate, using validation data and a learning rate decay of 0.8 for every 5 epochs. During training, a dropout rate of 0.3 was applied to the hidden layers to mitigate overfitting.^29^ Additionally, validation data errors were monitored to prevent overfitting and enable early stopping. The pixels were rescaled from 0–384 to 0–1 by dividing by 384. Furthermore, the pixel values were normalized using *Z*‐scores with a mean (0.5, 0.5, 0.5) and a standard deviation (0.1, 0.1, 0.1). All models were trained using batch‐normalized images. The training process encompassed 100 epochs, with a batch size of 50.

The optimal model, characterized by the lowest loss value, was selected for further analysis. Considering the imbalanced distribution of classes, particular emphasis was placed on the underrepresented examples. To achieve this, weights w1 = 0.7 and w0 = 0.3 were assigned to the LNM‐positive and ‐negative slides, respectively. This weighting scheme aimed to enhance the sensitivity of the models, particularly in detecting LNM‐positive slides.

### 2.7 Modal visualization and interpretability

In order to determine the relative significance of various tissue regions in predicting LNM at WSI level, patch scores were calculated for 384 × 384 patches (without overlap) within each tissue region. This computation was performed according to the reference method outlined in sections 2.5 and 2.6. To facilitate visualization, the scores of the patches were transformed to a range between 0.0 (indicating lower suspicion of metastasis) and 1.0 (indicating higher suspicion of metastasis) based on the initial score distribution. The interpretability model generated heatmaps, which were then converted to RGB values using a colormap and superimposed onto the original H&E images.^30^

### 2.8 Performance evaluation of our DL pipeline

In order to assess the efficacy of our predictor model, we employed various metrics such as sensitivity, specificity, Macro_F1_score, and AUC. These metrics were calculated using the scikit‐learn^31^ library in Python, which encompasses functions such as auc and classification_report.

### 2.9 Hardware and software

The raw WSIs were examined using Case‐Viewer, provided by 3DHISTECH, and uPath, provided by Roche. Image extraction and analysis were conducted using Pyvips (version 1.12.3) and OpenCV (version 4.1.1)^32^ in the Python programming language (version 3.6.6). Our high‐performance compute node on our main working platform utilized two NVIDIA Tesla A100 GPUs, each with 4GB of memory. The DL model training, validation, and testing were performed using PyTorch (version 1.2.0).^33^ For data estimation and visualization, Scikit‐learn (version 0.21.2) and Matplotlib (version 2.2.2) were employed in a collaborative manner.

## 3 RESULTS

### 3.1 Predict performance in internal test set

**FIGURE 4:**
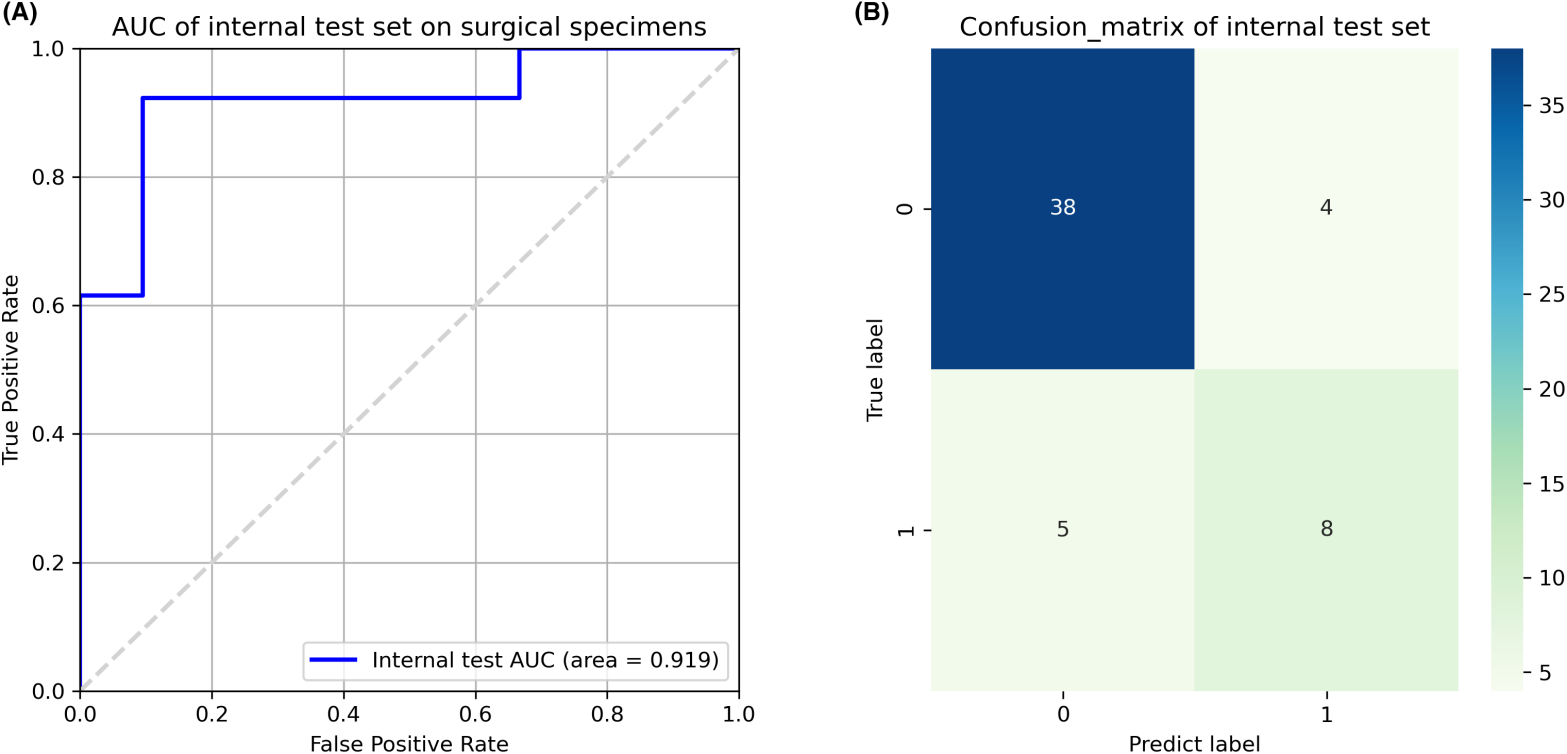
AUC (A) and confusion metrics (B) of internal test set.

The ViT network underwent training to extract features from patches, followed by the computation of a score for each patch. Subsequently, we selected the top‐K patches (where K = 10, 50, 100, 500, 1000, 1500). These K patches, which are deemed most representative, were then sequentially passed to the RNN for the purpose of predicting the final WSI level lymph node status. In the internal test set, the utilization of K = 500 resulted in the DL based digital‐pathological features predict LNM model producing a highly promising AUC of 0.919 (95% CI: 0.816–1.000). The sensitivity and specificity values were 0.923 (95% CI: 0.778–1.000) and 0.905 (95% CI: 0.816–0.994), respectively. Additionally, the accuracy (ACC) was determined to be 0.909 (95% CI: 0.906–0.912) as depicted in Figure 4 and Table 2.

**TABLE 2:** Metrics of internal testing set with variances Top‐K number of patches.

Top‐K (number of patches)						
Metrics	10	50	100	500	1000	1500
ACC (95% CI)	0.633 (0.601–0.665)	0.692 (0.658–0.727)	0.753 (0.715–0.791)	**0.909 (0.906–0.912)**	0.717 (0.681–0.753)	0.708 (0.673–0.743)
AUC (95% CI)	0.862 (0.819–0.905)	0.863 (0.820–0.906)	0.868 (0.825–0.911)	**0.919 (0.816–1.000)**	0.850 (0.808–0.893)	0.840 (0.798–0.882)
Sensitive (95% CI)	0.912 (0.866–0.958)	0.893 (0.849–0.938)	0.855 (0.812–0.898)	**0.923 (0.778–1.000)**	0.849 (0.807–0.892)	0.914 (0.868–0.960)
Specificity (95% CI)	0.812 (0.771–0.853)	0.832 (0.790–0.874)	0.880 (0.836–0.924)	**0.905 (0.816–0.994)**	0.851 (0.809–0.894)	0.766 (0.728–0.804)

The significance for bold values is to highlight that the model performs achieve best when the number of patches at 500.

### 3.2 Predict performance in external test set and biopsies test set

**FIGURE 5:**
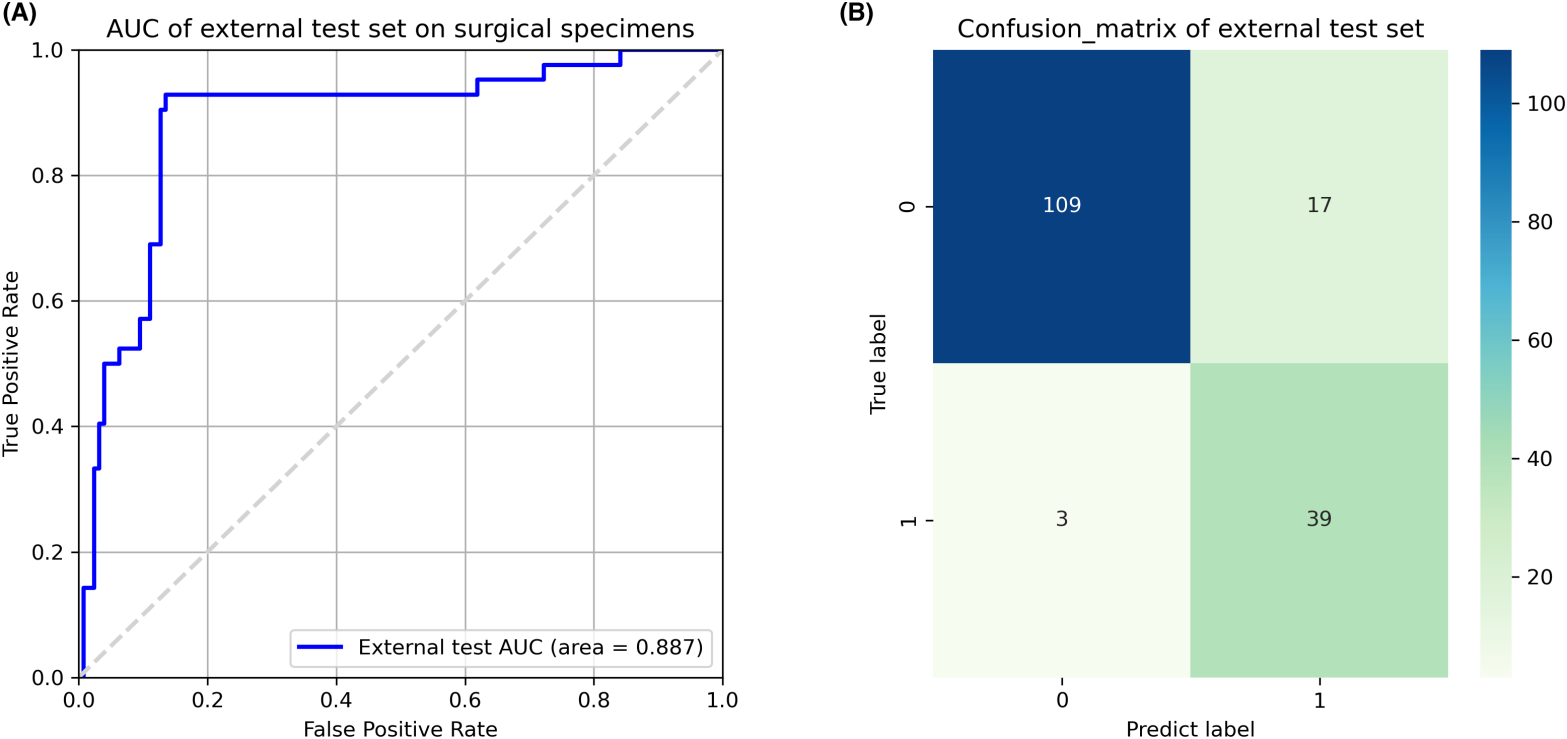
AUC (A) and confusion metrics (B) of external test set.

**FIGURE 6:**
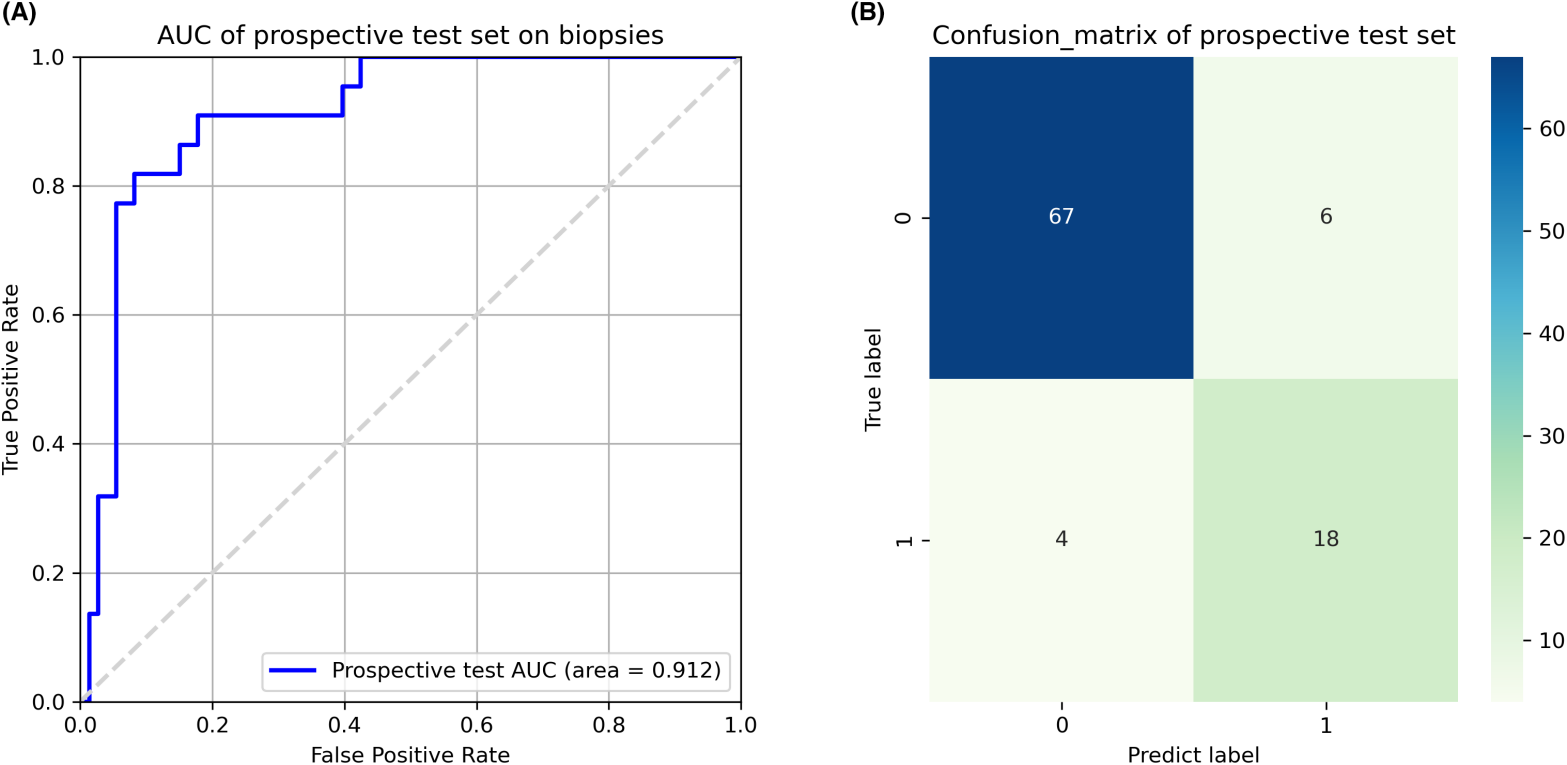
AUC (A) and confusion metrics (B) of biopsies test set.

In the external test set, after extracting features using Vit, we employed a selection process to identify the K most representative patches in each slide. Subsequently, these patches were sequentially inputted into the RNN. When K was set to 500, the DL model exhibited strong performance, achieving an AUC of 0.887 (95% CI: 0.823–0.950), sensitivity and specificity of 0.929 (95% CI: 0.851–1.000) and 0.865 (95% CI: 0.805–0.925), respectively. Additionally, the model demonstrated an ACC of 0.881 (95% CI: 0.880–0.882) (Figure 5, Table 3). In the cohort of biopsies in the test set, the number of patch features obtained from each WSI after removing blank patches is less than 500 due to the small size of the cervical biopsy pathology section. We selected all patches and inputted them into the RNN, resulting in the highest area under the curve (AUC) of 0.912 (95% CI: 0.849–0.976). The sensitivity and specificity were 0.818 (95% CI: 0.657–0.979) and 0.918 (95% CI: 0.855–0.981), respectively, while the accuracy (ACC) was 0.895 (95% CI: 0.893–0.897) (Figure 6).

**TABLE 3:** Metrics of external testing set with variances Top‐K number of patches.

Top‐K (number of patches)						
Metrics	10	50	100	500	1000	1500
ACC (95% CI)	0.672 (0.638–0.706)	0.691 (0.657–0.726)	0.734 (0.697–0.771)	**0.881 (0.880–0.882)**	0.736 (0.699–0.773)	0.715 (0.679–0.751)
AUC (95% CI)	0.807 (0.767–0.847)	0.808 (0.768–0.848)	0.811 (0.771–0.852)	**0.887 (0.823–0.950)**	0.822 (0.781–0.863)	0.765 (0.727–0.803)
Sensitive (95% CI)	0.832 (0.790–0.874)	0.812 (0.771–0.853)	0.750 (0.713–0.788)	**0.929 (0.851–1.000)**	0.749 (0.712–0.787)	0.763 (0.725–0.801)
Specificity (95% CI)	0.781 (0.742–0.820)	0.803 (0.763–0.843)	0.872 (0.828–0.916)	**0.865 (0.805–0.925)**	0.895 (0.850–0.940)	0.766 (0.728–0.804)

The significance for bold values is to highlight that the model performs achieve best when the number of patches at 500.

### 3.3 Diagnostic performance of radiography

In the Qilu cohort, the radiography imaging examination demonstrated a sensitivity of 0.528 (95% CI: 0.502–0.554) and a specificity of 0.789 (95% CI: 0.750–0.828), with an AUC of 0.659 (95% CI: 0.528–0.789). Similarly, in the prospective cohort, the radiography imaging examination showed a sensitivity of 0.474 (95% CI: 0.450–0.498) and a specificity of 0.917 (95% CI: 0.871–0.963), with an AUC of 0.695 (95% CI: 0.474–0.917).

### 3.4 Model interpretation: visualizing predictions with heatmaps

**FIGURE 7:**
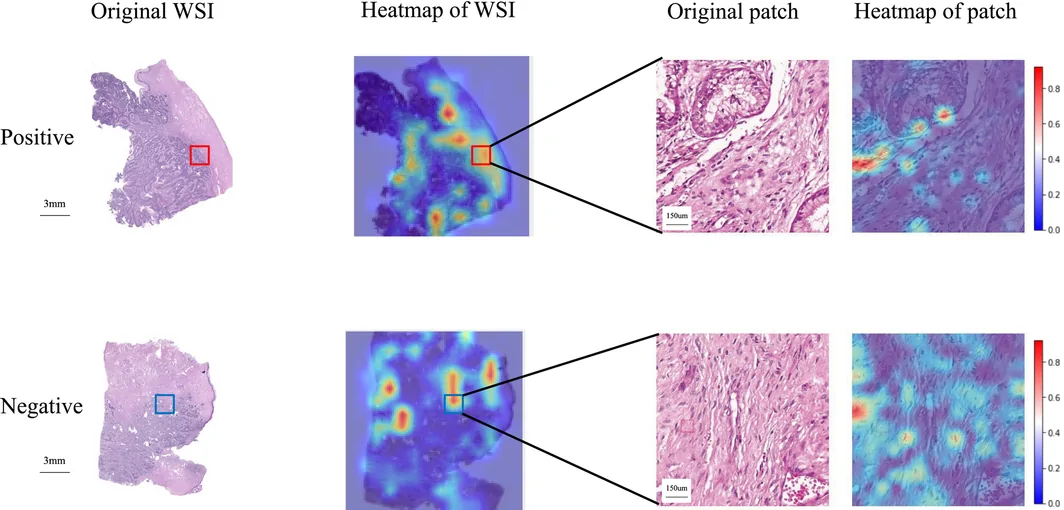
Visualization heatmaps of tissue predictions of LNM‐positive and LNM‐negative slide, respectively.

In order to elucidate the utilization of WSIs for the prediction of LNM, our model was subjected to interpretation and subsequent validation. This involved the computation of relevance scores based on layer‐wise relevance propagation (LRP) for each attention head and propagation through all layers. By quantifying the scores of patches, heatmaps were generated within the WSIs and subsequently superimposed onto the original H&E stained tissue sections. Our investigation focused on the regions of utmost significance within the WSIs, specifically within the top 10% (LNM‐positive group) and bottom 10% (LNM‐negative group) of predicted LNM risks for patients. These regions serve as indicators of support and nonsupport for the occurrence of LNM. In general, we observed that high‐significance regions in LNM‐negative patients were characterized by a higher presence of immune cells (17.63% vs. 11.44%, two‐sided Mann–Whitney *U*‐Test, *p* < 0.05) and lower tumor grade compared to those in LNM‐positive patients. Furthermore, our observations indicate a correlation between high‐relevance regions in patients with LNM and a higher presence of tumor cells (72.21% vs. 63.98%, as determined by a two‐sided Mann–Whitney *U*‐test with a significance level of *p* < 0.05), as well as increased invasion, as depicted in Figure 7.

## 4 DISCUSSION

Through the analysis of histopathology slides from patients diagnosed with cervical cancer, we successfully anticipated the presence of LNM by examining surgical specimens obtained post‐operation. Furthermore, we validated this predictive capability by accurately assessing biopsied tissue obtained pre‐operation. Notably, this study represents the pioneering use of DL techniques for LNM prediction in cervical cancer patients, specifically utilizing histopathology biopsies obtained prior to surgery.

It is advisable for women diagnosed with early‐stage cervical cancer (FIGO stage IA2‐IIA2) to undergo a primary radical hysterectomy in conjunction with a systemic pelvic lymphadenectomy. The incidence of pelvic LNM in patients with early‐stage cervical cancer varies between 15.0% and 20.0%, and significantly impacts disease recurrence and mortality, thereby impacting therapeutic schedule choices for patients.^3^, ^5^ The primary modification in the 2018 FIGO staging system pertains to the categorization of patients as “stage IIIC” upon confirmation of LNM through imaging or pathological findings. Despite the growing significance of LNM status, the optimal radiological approach for detecting LNM remains uncertain. Furthermore, numerous clinicians have indicated that clinical examination does not consistently align with pathological outcomes, as evidenced by the need for therapeutic adjustments in over 20%–40% of patients following surgical assessment of lymph nodes concerns.^34^ For early‐stage cervical cancer patients, the inclusion of lymph node dissection during surgery not only prolongs the duration of the procedure and results in heightened intraoperative blood loss, but also gives rise to a multitude of severe complications, including lymphatic cysts, infections, venous thromboembolism, vascular and nerve impairment, as well as substantial pelvic and abdominal adhesion. Therefore, it is crucial to explore strategies that minimize the need for systematic lymph node dissection while preserving the efficacy of tumor treatment. Hence, the accurate prediction of lymph node status prior to surgery and subsequent implementation of tailored treatment strategies for patients with varying risks of LNM are imperative clinical concerns.

Pathology serves as a fundamental pillar in contemporary medicine, particularly in the realm of cancer diagnosis and treatment. The diagnostic expertise of pathologists holds paramount importance in both clinical and pharmaceutical research, as well as in the determination of appropriate patient treatment. The evaluation of tumor malignancy by pathologists' hinges upon meticulous examination of histological sections under a microscope, wherein the degree of cell atypia and growth pattern are observed. However, it is worth noting that pathologists, being human, may experience fatigue and inadvertently overlook crucial information during their microscopic analyses. In recent years, the advancement of computer technology has facilitated the utilization of scanners to convert histological sections into WSIs, thereby aiding pathologists in their diagnostic processes. This integration of computer technology has the potential to enhance the efficiency and accuracy of pathologists, ultimately leading to improved patient treatment outcomes. Furthermore, the significant progress made in artificial intelligence (AI) has had a profound global impact over the past few decades. Particularly within the field of medicine, the application of AI in diagnostics, including radiology and pathology, has demonstrated promising outcomes.^35^, ^36^, ^37^ Histopathological tissue sections, include detecting and categorizing tumors, have the potential to use AI analysis, such as segmentation of images,^38^ detecting and counting cells,^39^ detecting mitosis,^40^ analysis of kidney transplant biopsy samples, and grading of tumors are some of the applications.^41^, ^42^ It is currently handled by specialized experts, pathologists who carefully assess gigapixel images.^43^, ^44^

In recent years, there are also some researches detect lymph node status from lymph node tissue obtained in post‐operation such as colorectal cancer micro‐metastasis,^45^ metastasis of lymph nodes in lung cancer,^46^ metastases to lymph nodes in prostate cancer,^47^ gastric cancer lymph node quantification and metastatic,^48^ melanoma tumors sentinel lymph node status,^49^ however, there is a lack of pertinent research on cervical cancer. The aforementioned studies exclusively utilized histopathological tissue from postoperative lymph nodes, rather than primary tumor specimens or biopsies, thereby rendering them inadequate for informing preoperative therapeutic decisions for clinicians.

In both the external cohort and prospective cohort, we incorporated patients from multiple centers and utilized two scanners. These distinct patient cohorts and scanners exhibit substantial technical variability. By employing our developed DL model, we observed a decrease in the AUC to 0.887 in the external cohort and 0.912 in the prospective cohort, when compared to the internal test set. Nevertheless, these results remain significantly superior, this observation implies that the model acquired biologically significant features from the Qilu cohort's training and validation sets, rather than merely identifying coincidental artifacts that are associated with LNM. However, the diminished performance exhibited by the model highlights the inherent limitation of deep learning‐based image classifiers in effectively generalizing to slides originating from diverse sources, which may possess slight variations in staining, scanning, and other technical attributes.

In the prospective cohort study, a total of 79 patients underwent radiographic imaging examinations, with 54 of them specifically undergoing MRI examinations. Among these 54 patients, 14 were diagnosed with LNM post‐operation. Of these, 12 were correctly identified using a DL model, while 6 received accurate diagnoses through MRI, importantly, all these 6 patients were correctly diagnosed DL model, indicating the superior sensitivity of the model in detecting patients identified by MRI, as well as additional patients that MRI alone could not identify.

Furthermore, by employing heatmaps, one can effectively identify latent histopathological patterns, thereby facilitating the interpretation of outcomes. Sytse J et al. have documented that cervical cancer cases exhibiting elevated levels of infiltrating T cells, specifically CD4^+^ and CD8^+^ cells, demonstrate a higher CD8^+^/CD4^+^ T cell ratio in patients without LNM compared to those with LNM.^50^ Consistently across all patients, a notable presence of immune cells and lower tumor grade was observed in high‐relevancy regions within LNM‐negative tumors, in comparison to those with LNM‐positive tumors. Furthermore, our observations indicate that high‐relevancy regions are linked to heightened tumor cell presence and invasion in LNM‐positive patients, both of which are recognized as higher risk factors for LNM. These findings strongly suggest that our model successfully acquired the ability to extract biologically significant features from primary tumor lesions.

## 5 LIMITATIONS AND FUTURE RESEARCH

Initially, it is imperative to ensure a sufficiently larger training sample size for the DL model. Consequently, incorporating additional WSIs into our DL model training would prove advantageous. Additionally, the model was initially trained using surgical specimens acquired post‐operation. This discrepancy in sample types diminishes the value and clinical implications of preoperative prediction of LNM, and now we are working on training the model with biopsies obtained pre‐operation, and integrate clinical‐pathological features (such as patients' age, tumor size, FIGO stage, histology subtype, differentiation, HPV infection, SCC‐Ag, etc.), to predict LNM in pre‐operation. Furthermore, despite the potential shown by this result, the utilization of DL algorithms in clinical settings remains limited. The need to strike a balance between the exaggerated expectations and the optimistic prospects associated with new techniques necessitates the undertaking of extensive prospective clinical trials encompassing a sufficient number of WSIs. These trials are imperative in order to validate the aforementioned findings and substantiate the genuine advantages of AI in accurately predicting LNM prior to surgical intervention.

## 6 CONCLUSION

In summary, we have successfully developed a model capable of acquiring pertinent histopathological characteristics associated with LNM in patients with cervical cancer, utilizing primary tumor specimens' H&E images. Furthermore, we have validated the model's efficacy in accurately predicting lymph node positivity through preoperatively obtained biopsies. Our research findings demonstrate the efficiency of a Vit and RNN DL model in this regard. The preoperative implementation of this model exhibited promising results in potentially enhancing the accuracy of lymph node status detection and facilitating the administration of the most optimal treatment for patients. This, in turn, offers valuable insights for the development of individualized treatment strategies, such as performing surgery exclusively involving radical hysterectomy or radical cervical resection for patients with a very low risk of LNM, thereby avoiding unnecessary lymphadenectomy and its associated complications for 80.0%–85.0% early‐stage cervical cancer patients; and if the patient exhibits a significantly elevated risk of LNM or the presence of metastasis can be detected, radical chemoradiotherapy is the recommended treatment option, thereby mitigating potential surgical complications for 15.0%–20.0% cervical cancer patients.

## AUTHOR CONTRIBUTIONS

**Qingqing Liu:** Methodology (equal); writing – original draft (equal). **Nan Jiang:** Methodology (equal); writing – original draft (equal). **Yiping Hao:** Methodology (equal); writing – original draft (equal). **Chunyan Hao:** Resources (equal). **Wei Wang:** Data curation (equal). **Tingting Bian:** Data curation (equal). **Xiaohong Wang:** Data curation (equal). **Hua Li:** Data curation (equal). **Yan Zhang:** Data curation (equal). **Yanjun Kang:** Data curation (equal). **Fengxiang Xie:** Data curation (equal). **Yawen Li:** Data curation (equal). **Xuji Jiang:** Validation (equal); visualization (equal). **Yuan Feng:** Validation (equal); visualization (equal). **Zhonghao Mao:** Formal analysis (equal). **Qi Wang:** Data curation (equal). **Qun Gao:** Data curation (equal). **Wenjing Zhang:** Data curation (equal). **Baoxia Cui:** Conceptualization (equal); funding acquisition (equal); project administration (equal); supervision (equal); writing – review and editing (equal). **Taotao Dong:** Conceptualization (equal); software (equal); supervision (equal); writing – review and editing (equal).

## FUNDING INFORMATION

This work was supported by the Innovation and Development Joint Funds of Natural Science Foundation of Shandong Province (ZR2021LZL009) to Taotao Dong. Clinical Research Center of Shandong University (No.2020SDUCRCA007) to Baoxia Cui.

## CONFLICT OF INTEREST STATEMENT

The authors declare that there is no conflict of interest.

## ETHICAL APPROVAL

Sensitive information such as the patient's name, the medical record number, and ID number for this research project were anonymized to protect patients' privacy and the ethics committees at each hospital approved this research before it was initiated (approval number KYLL‐202022‐080).

## Data Availability

All other data generated from this study are available upon request to the corresponding author. We used code can freely available at https://github.com/huggingface/pytorch‐image‐models/tree/main/timm.
